# Hospital *Enterococcus faecium* demonstrates distinct environmental and patient reservoirs: a genomic point prevalence survey

**DOI:** 10.1017/ice.2025.27

**Published:** 2025-05

**Authors:** Nenad Macesic, Hugh Cottingham, Jessica A. Wisniewski, Luke V. Blakeway, Ravali Theegala, Katherine Pragastis, Andrew Stewardson, Pauline Bass, Megan Gritt, Stephanie Spilsbury, Denise Del Rosario-Kelly, Amanda Dennison, Denis W. Spelman, Adam W.J. Jenney, Anton Y. Peleg

**Affiliations:** 1 Department of Infectious Diseases, The Alfred Hospital and School of Translational Medicine, Monash University, Melbourne, Australia; 2 Infection Prevention & Healthcare Epidemiology, Alfred Health, Melbourne, Australia; 3 Centre to Impact AMR, Monash University, Clayton, Australia; 4 Microbiology Unit, Alfred Health, Melbourne, Australia; 5 Infection Program, Monash Biomedicine Discovery Institute, Department of Microbiology, Monash University, Clayton, Australia

## Abstract

We assessed the hospital environment as a reservoir of vancomycin-resistant *E. faecium* (VRE) and compared environmental VRE isolates to bloodstream infection *E. faecium* isolates. We identified distinct environmental and patient reservoirs, with the environment dominated by *vanB* VRE. Environment-clinical reservoir spillover accounted for 292/895 (33%) of putative transmission links.

## Introduction

*Enterococcus faecium* is a common cause of hospital-acquired infections and is frequently associated with vancomycin resistance, resulting in high mortality, longer hospital stays, and higher healthcare costs.^1^ The hospital environment is a reservoir for *E. faecium* due to its ability to survive on surfaces for prolonged periods.^1^ Prior studies have analyzed patient and environmental reservoirs of *E. faecium* but have typically been limited to selected hospital wards in settings where either *vanA* or *vanB* vancomycin-resistant *E. faecium* (VRE) predominate.^2–4^ Australia has a specific VRE epidemiology with more recent emergence of *vanA* VREfm on a background of *vanB* VRE endemicity, with both continuing to circulate.^5^ In 2019, we noted increasing prevalence of *vanA* VRE bloodstream infections. We aimed to assess the hospital environment as a reservoir of *vanA* VRE by conducting a point prevalence study and comparing environmental and contemporary clinical isolates using genomic analyses.

## Methods

The study was approved by the Alfred Hospital Ethics Committee (Project Number 81/24, low-risk pathway). The Alfred Hospital (Melbourne, Australia) is a 600-bed quaternary hospital with state referral services for burns and trauma, as well as stem cell and solid organ (heart/lung/kidney) transplant services. We conducted a point prevalence survey of the hospital environment. We sampled surfaces using FLOQswabs (Copan) over two months (see Supp. Methods and Supp. Table 1), which included all wards and the intensive care unit (ICU) (n = 12). Each ward was sampled once during that two-month period. To minimize disruption to patient care and staff workflows, within individual units, we focused on equipment and rooms that were not in active use at the time of sampling.

**Figure 1.**
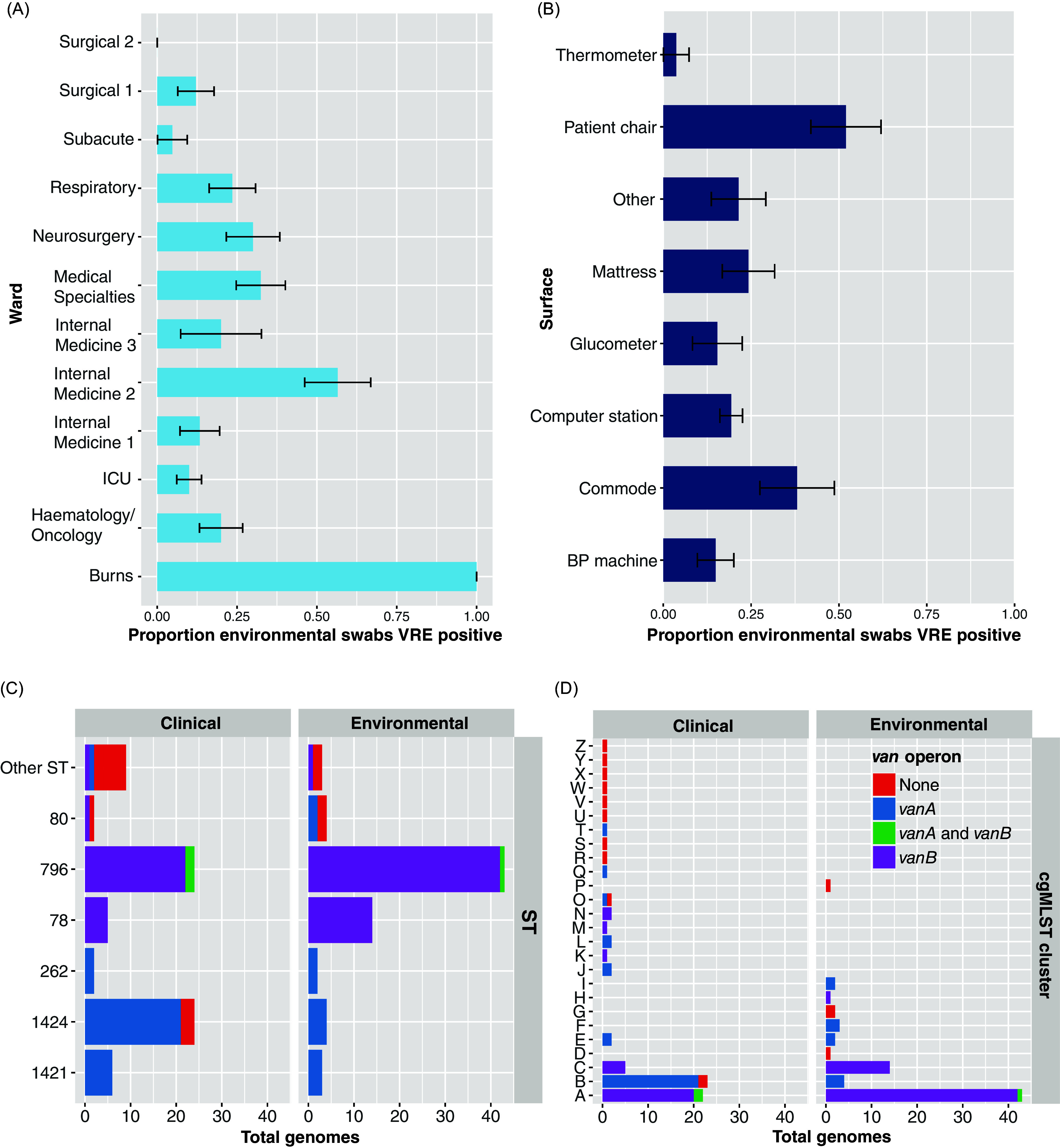
Summary of *Enterococcus faecium* environmental screening and contemporary clinical isolates. (A) Proportion of environmental screening swabs positive for vancomycin-resistant *E. faecium* (VRE) by ward and (B) by surface type. ‘Other’ includes bedside monitors, trolleys, and call bells. (C) Summary of *E. faecium van* operon presence by multi-locus sequence types (MLST) and (D) core genome MLST (cgMLST) in clinical and environmental isolates.

Ten surface types were sampled across the hospital (see Figure 1B, Supp. Table 2) with multiple samples of each surface type collected across different wards. Swabs were premoistened with sterile saline and used to sample a 10 × 10 cm area of each surface. After incubation in enrichment broth, samples were cultured on CHROMagar VRE media (BioMerieux) and species were identified using matrix-assisted laser desorption-ionization time-of-flight (MALDI-TOF) (Bruker Daltonics). All *E. faecium* isolates were presumed to be VRE but no phenotypic antimicrobial susceptibility testing was performed.

To assess for molecular epidemiological links between environmental and clinical isolates, we selected all *E. faecium* isolates from our established blood culture biobank (approved by Alfred Hospital Ethics Committee, Project Number 533/16, biobank pathway), which comprehensively stores all blood culture isolates from our hospital. Phenotypic antimicrobial susceptibility testing data were not available. We included all *E. faecium* isolates collected during the period starting 6 months before the first environmental sampling date and ending 6 months after the final environmental sampling date (Supp. Fig. 1 and Supp. Table 2). Clinical data were extracted from the electronic medical record. Our hospital does not perform asymptomatic screening for VRE. See Supp. Methods for details of routine environmental cleaning and infection prevention practices. In brief, surfaces were cleaned daily with 10% sodium hypochlorite solution with some high-risk high-touch surfaces cleaned more frequently in extreme-risk wards.

One colony was selected from each sample and all environmental and clinical isolates from the study period underwent short-read (Illumina) whole genome sequencing, as described previously.^6^ We detected *van* operon presence and assigned multi-locus sequence type (MLST) and core genome MLST (cgMLST).^7^ For each cgMLST cluster, we assembled a long-read reference genome and calculated within-cluster pairwise single nucleotide variant (SNV) distances (see Supp. Methods).

## Results

### Environmental point prevalence survey

We swabbed 357 surfaces (median 31.5 per ward [range 10–60]). A total of 73/357 (20%) surfaces isolated VRE, with median 20% positive per ward (IQR 11.6%–30.6%) (Figure 1A, Supp. Table 2), indicating that VRE was endemic in the hospital environment. Rates of VRE positivity varied widely, with the highest rate in the Burns ward (10/10 swabs positive) and the lowest rate in Surgical Ward 3 (0/34 positive). Patient chairs had the highest rate of VRE positivity (13/25 [52%]) (Figure 1B, Supp. Table 3).

### Environmental and clinical genome characteristics

All 72 *E. faecium* blood culture isolates from the study period were sequenced and compared to the 73 positive environmental swabs (Figure 1C, Supp Fig. 1). Of 72 blood culture isolates, 63 (88%) were hospital–onset (collected >48 hours after hospital admission). *vanA* and *vanB* were detected in 12/73 (16%) and 58/73 (79%) environmental genomes, respectively (Figure 1C). In contrast, 32/72 (44%) clinical genomes carried *vanA* (*P* <.001, χ^2^ test), and 31/72 (43%) carried *vanB*. Environmental genomes belonged to 10 MLSTs and 10 cgMLSTs, with ST796/ST78 accounting for 54/73 (74%) genomes (Figure 1C and 1D). Clinical STs/cgMLSTs overlapped with environmental genomes but were more diverse. Specifically, ST1421/1424 carried *vanA* and were more frequent in clinical genomes (30/72 v 7/73 genomes, *P* <.001, χ^2^ test). A single cgMLST cluster (B) contained the majority of *vanA* genomes (25/44 genomes), while *vanB* genomes were noted across two cgMLST clusters (A and C, 84/89 genomes) (Figure 1D). STs/cgMLSTs carried either *vanA* or *vanB* operons, except for 3 genomes in ST796/cgMLST cluster A that had concurrent *vanA* and *vanB* carriage (Figure 1C/1D).

### Geography of E. faecium on hospital wards

We mapped *E. faecium* ST/cgMLST and presence of *van* operon across individual wards (Supp. Fig. 2). High-risk wards (ICU, Haematology/Oncology) had the majority of clinical genomes with a resultant high diversity of STs/cgMLSTs (7 STs/10cgMLSTs for Haematology/Oncology and 7 STs/7 cgMLSTs for ICU, respectively). The Haematology/Oncology ward had the highest proportion of *vanA* genomes (12/25, 48%), with 7/12 of these being ST1424/cgMLST cluster B genomes. Other wards had uniform ST/cgMLST composition with no differences between wards (Supp. Fig. 2).

### E. faecium transmission networks

**Figure 2.**
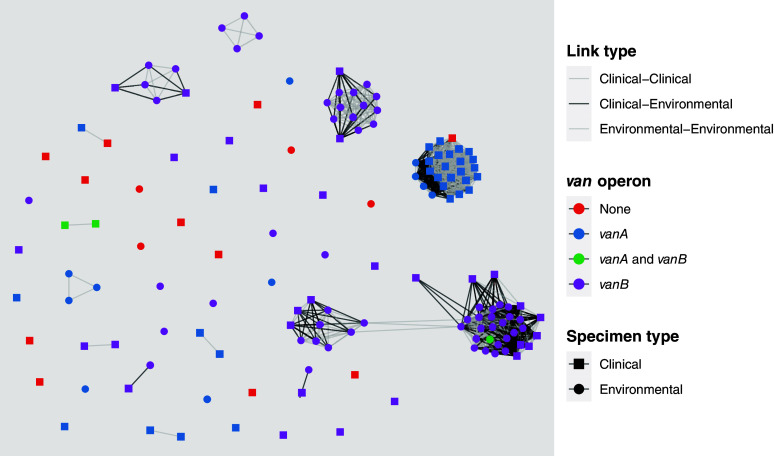
Network analysis of *Enterococcus faecium* putative genomic transmission links. Clinical and environmental *E. faecium* genomes are shown as nodes and putative genomic transmission events (defined as pairwise single nucleotide variant distance ≤6) as edges. Edges resulting from clinical-environmental links are shown in black, and clinical-clinical or environmental-environmental in gray. A single major *vanA* cluster is noted with majority of clinical genomes. In contrast, three major *vanB* clusters are noted with environmental genomes predominating.

Using a 6 SNV cutoff,^3^ there were 895 putative genomic transmission links resulting in 13 clusters (Figure 2). For genetically-related isolates, median time between isolate collection dates was 37 days (IQR 14–124) (Supp. Fig. 3). While 292/895 (33%) links were environmental-clinical, of these only 88/292 (30%) were between *vanA* genomes. Most clusters had a predominance of either environmental-environmental links or clinical-clinical links (Supp. Table 5). A single cluster contained 25/44 (57%) *vanA* genomes, with the majority being clinical (22/26, 85%). Environmental genomes in that cluster came from three wards, while clinical genomes were collected from patients in 11 different wards. In contrast, *vanB* genomes belonged to three major (>5 genomes) clusters, containing predominantly environmental genomes (42/56, 75%).

### van operon analysis

In completed assemblies (n = 26), the *vanA* and *vanB* operons were located on pRE25-like plasmids and the chromosome, respectively. The pRE25-like plasmids were heterogeneous and did not indicate spread of a single plasmid across multiple cgMLST clusters (Supp. Fig. 4).

## Discussion

We noted a 20% prevalence of VRE environmental colonization across a diverse range of hospital surfaces, but only 12/73 (17%) environmental genomes carried *vanA*. This indicated that while VRE was endemic in the hospital environment, it likely did not make a major contribution to the increased *vanA* VRE prevalence in bloodstream infection isolates. Indeed, we noted distinct environmental and patient reservoirs: an environment dominated by *vanB* ST796/78 genomes, and a clinical reservoir comprising *vanA* ST1421/1424 genomes. There was evidence of spill-over with putative clinical-environmental links, but these were a minority (33%) and *vanA* putative genomic links only contributed 88/895 (10%) of these in turn.

Our findings stand in contrast to prior work showing significant links between VRE in the environment and clinical colonization and infection.^2–4^ This difference may be due to these studies describing non-outbreak settings and focusing on limited wards (ICU and Haematology/Oncology). Potential contributors to our findings include our institution’s differing infection prevention approaches to *vanA* and *vanB* VREfm-colonized patients:^8,9^ *vanA* patients are routinely placed in contact precautions with dedicated equipment, while for *vanB* patients this occurs only on high-risk wards, and if they have diarrhea or non-contained wounds. The environmental isolates in our study may represent persistent strains of VRE. *E. faecium* ST796, a key *vanB* lineage in our study, has been shown to develop biocide tolerance that may provide it with a fitness advantage in hospital environments.^10^ Of note, the *vanA* clinical reservoir that prompted our investigation was subsequently resolved during the COVID-19 pandemic (Supp. Fig. 1), perhaps because of these factors.

Our study had several limitations. To assess the clinical reservoir, we focused on bloodstream infection isolates. However, these likely represent the ‘tip of the iceberg’ of VRE colonization with only 8% of VRE-colonized patients developing infection.^11^ Similarly, our institution does not conduct asymptomatic VRE screening, limiting our ability to quantify rates of VRE colonization in patients. We also included all *E. faecium* bloodstream infection culture isolates (both VRE and non-VRE), potentially impacting comparison with the environmental VRE isolates. However, 68/72 (94.4%) clinical isolates carried the *van* operon, thus limiting this impact. Sampling equipment and rooms not in active use were a potential source of bias, however, this pragmatic approach allowed us to conduct sampling efficiently while avoiding interference with clinical activities. We also selected single colonies for whole genome sequencing, which may not be representative of the entire enterococcal population, particularly for environmental samples. Finally, our analysis of transmission networks was limited by the absence of patient movement data.

Although the environment plays a major role in healthcare-associated VRE spread, our study shows that distinct clinical and environmental reservoirs of VRE may exist, particularly in outbreak settings where new clones are emerging and have not yet colonized the environment. This provides support for use of molecular techniques to focus infection prevention strategies according to the reservoir. While cleaning strategies aiming to reduce the environmental burden form an essential part of any multifaceted approach to VRE control,^12^ the clinical reservoir may require more active attention through measures such as introduction of active surveillance, redoubled hand hygiene efforts, and more stringent application of contact precautions in order to prevent both between-patient spread and future environmental colonization.

## Supporting information

Macesic et al. supplementary material Macesic et al. supplementary material

## Supplementary material

The supplementary material for this article can be found at https://doi.org/10.1017/ice.2025.27

## Conference presentation

Portions of this work were presented at European Society of Clinical Microbiology and Infectious Diseases Global 2024, held in Barcelona, Spain.
