# Supplementary material for: Hospital Enterococcus faecium demonstrates distinct environmental and patient reservoirs: a genomic point prevalence survey
Source: Infect Control Hosp Epidemiol. 2025 Mar 21;46(5):540–3. doi: 10.1017/ice.2025.27 (PMC12034451; doi:10.1017/ice.2025.27)
Supplement: Macesic et al. supplementary material [file S0899823X25000273sup001.pdf]

Table of contents

Supplementary Methods....2
References....6
Supp. Fig. 1 – *Enterococcus faecium* clinical bloodstream isolate collection....7
Supp. Fig. 2 – Summary of *Enterococcus faecium* environmental and clinical genomes by ward....8
Supp. Fig. 3 – Time between collection dates of genetically-related isolates....9
Supp. Fig. 4 – Plasmids with *van* operons from *Enterococcus faecium* completed assemblies....10
Supp. Table 1 – Summary of included clinical and environmental *Enterococcus faecium* genomes...11
Supp. Table 2 – Environmental surface types sampled during study....16
Supp. Table 3 – Summary of environmental swabs positive for vancomycin-resistant *Enterococcus faecium*....16
Supp. Table 4 – Summary of surface types positive for vancomycin-resistant *Enterococcus faecium* 16
Supp. Table 5 – Summary of putative *Enterococcus faecium* genomic transmission clusters....17

Supplementary Methods

Environmental cleaning

Environmental cleaning was occurring daily as per the Australian Guidelines for the Prevention and Control of Infection in Healthcare[1] (see Table A2.2). According to these guidelines, hospital wards are classified as high risk (e.g. general wards) or extreme risk (e.g. ICUs or wards with immunosuppressed patients). Our study included both high and extreme risk wards. Daily cleaning occurred in both settings, with some high-risk high-touch surfaces cleaned more frequently in extreme risk wards. A 10% sodium hypochlorite solution was used for cleaning.

Environmental sampling

Environmental sampling was done using the following protocol:

Sampling

1. Pre-moisten FLOQswab (Copan) with sterile saline.
2. Sample a 10 cm x 10 cm area or the maximum area possible.
3. Sample areas and methods must be consistent for similar sites or objects.
4. Transport swabs immediately to microbiology laboratory.

Bacterial enrichment and primary isolation

1. Prepare 15mL Falcon tubes containing 2mL Heart Infusion (HI) broth.
2. Break FLOQswab shaft and place swab head into tube containing HI broth.
3. Vortex tube for 10 secs, remove swab head and discard.
4. Grow at 37°C with shaking at 200rpm until turbid (max 72 hours).
5. Transfer 400µL of suspension to 2mL cryovial and add 600µL of 50% glycerol in Brain Heart Infusion (final stock concentration = 30% glycerol).
6. Store glycerol stock at -80°C.
7. Streak 10µL of suspension on a CHROMagar VRE plate using a 10µL loop (half plate per sample).
8. Grow overnight at 37°C.

Secondary streaking and storage of pure cultures

1. Pick 6 single colonies and streak on HI agar (half plate per colony).
2. Grow overnight at 37°C.
3. Stock isolate in 1ml Brain Heart Infusion (BHI) broth with 30% glycerol in 2mL cryovial.
4. Store glycerol stock at -80°C.
5. Take 2-3 colonies and proceed with matrix-assisted laser desorption-ionisation time-of-flight (MALDI-TOF) identification.

Culture, DNA extraction and sequencing

All stored bacterial isolates were grown on BHI agar (Becton-Dickinson) at 37°C for 16 hours. Genomic DNA was extracted from bacterial plate culture using the GenFind V3 Reagent Kit (Beckman Coulter) as per manufacturer's instructions. Libraries for short read sequencing were prepared using the Nextera Flex DNA Library Prep Kit (Illumina), and 150 bp paired-end sequencing was performed on the NovaSeq 6000 system (Illumina). Libraries for long-read sequencing were prepared using the Ligation Sequencing Kit with Native Barcoding Expansion (Oxford Nanopore Technologies) and sequenced on the GridION instrument with an R10.4.1 flow cell (Oxford Nanopore Technologies) for 48 hours. Basecalling was performed with Guppy v.4.0.14 using the 'super' basecalling model.

De novo assembly and annotation

We constructed *de novo* assemblies of all isolates with only short-read data using Unicycler[2]. Assembly quality was checked using Quast v5.2.0[3] and species identification was performed using GTDB-Tk v1.0.2[4]. Genomes were annotated using Prokka v1.14.6[5]. We then performed resistance gene and plasmid replicon detection with Abricate v.1.0.0[6], using the NCBI Antibiotic Resistance and PlasmidFinder databases, respectively. We determined *in silico* multi-locus sequence type (ST) using 'mlst' v.2.19.0[7].

Core genome multi-locus sequence typing (cgMLST) and related analyses

We defined cgMLST of each isolate using the approach of Higgs et al.[8]. In brief, we assigned cgMLST alleles using the public *Enterococcus faecium* cgMLST scheme[9] (core set of 1,423 genes) and

chewBBACA (v2.0.16)[10]. We then determined the number of allelic differences between each genome and assigned cgMLST clusters using single linkage clustering and a pairwise allelic difference threshold of ≤25.

For each cgMLST cluster with ≥2 genomes, we then selected the earliest available isolate as a reference and generated long-read sequencing data as described above. We then constructed *de novo* assemblies incorporating short- and long-read data for these isolates. We used a long-read-first assembly approach using a bespoke pipeline (https://github.com/HughCottingham/clinopore-nf) that incorporates Flye v2.9.2 with subsequent polishing with Medaka v1.8.0, Polypolish v0.5.0 and Polca v3.4.1[11-15].

Using these completed assemblies as references, we generated a core chromosomal single nucleotide variant (SNV) alignment using Snippy v.4.6.0 [16] for each cgMLST. We then calculated pairwise SNV distances for all genomes in that cgMLST. Genomes with ≤6 SNV differences were considered putative genomic transmission events [17]. We then used the R package 'ggraph' v2.0.5 to visualize putative transmission networks with genomes as nodes and putative genomic transmission events as edges.

Plasmid analyses

Using Abricate, we identified *van* operon-harboring contigs that were putative plasmids in our reference completed assemblies. We used progressiveMauve v2.4.0.r4736 to align all plasmids and assess for structural re-arrangements [18]. Geneious Prime 2023.2.1 (https://www.geneious.com) was used to visualize and assess for structural re-arrangements, with subsequent manual annotation in Adobe Illustrator v2020.24.3.

Data availability

Illumina/Nanopore read data were deposited in the NCBI Sequence Read Archive under project accession PRJNA1077967. Completed genome assemblies were deposited in GenBank; accessions are listed in Supp. Table 1.

Statistical analysis

Categorical variables were compared using $\chi 2$ or Fisher's exact tests and continuous variables were compared using Student’s *t*-test or Mann-Whitney-Wilcoxon, as appropriate. Statistical analyses were performed in R (v4.3.0).

References

1. National Health and Medical Research Council. Australian Guidelines for the Prevention and Control of Infection in Healthcare. In: Commonwealth of Australia, ed. Canberra, Australia2019.

2. Wick RR, Judd LM, Gorrie CL, Holt KE. Unicycler: Resolving bacterial genome assemblies from short and long sequencing reads. *Plos Comput Biol* 2017;13:e1005595.

3. Gurevich A, Saveliev V, Vyahhi N, Tesler G. QUAST: quality assessment tool for genome assemblies. *Bioinformatics* 2013;29:1072-1075.

4. Chaumeil PA, Mussig AJ, Hugenholtz P, Parks DH. GTDB-Tk: a toolkit to classify genomes with the Genome Taxonomy Database. *Bioinformatics* 2019;36:1925-1927.

5. Seemann T. Prokka: rapid prokaryotic genome annotation. *Bioinformatics* 2014;30:2068-2069.

6. *Abricate: mass screening of contigs for antimicrobial resistance or virulence genes* [computer program]. 2020.

7. *mlst: Scan contig files against traditional PubMLST typing schemes* [computer program]. 2020.

8. Higgs C, Sherry NL, Seemann T, et al. Optimising genomic approaches for identifying vancomycin-resistant Enterococcus faecium transmission in healthcare settings. *Nat Commun* 2022;13:509.

9. de Been M, Pinholt M, Top J, et al. Core Genome Multilocus Sequence Typing Scheme for High- Resolution Typing of Enterococcus faecium. *J Clin Microbiol* 2015;53:3788-3797.

10. Silva M, Machado MP, Silva DN, et al. chewBBACA: A complete suite for gene-by-gene schema creation and strain identification. *Microb Genom* 2018;4:e000166.

11. Wick RR, Schultz MB, Zobel J, Holt KE. Bandage: interactive visualization of de novo genome assemblies. *Bioinformatics* 2015;31:3350-3352.

12. Kolmogorov M, Yuan J, Lin Y, Pevzner PA. Assembly of long, error-prone reads using repeat graphs. *Nat Biotechnol* 2019;37:540-546.

13. Wick RR, Holt KE. Polypolish: Short-read polishing of long-read bacterial genome assemblies. *Plos Comput Biol* 2022;18:e1009802.

14. Wick RR, Judd LM, Holt KE. Assembling the perfect bacterial genome using Oxford Nanopore and Illumina sequencing. *SciELO Preprints* 2022.

15. Zimin AV, Salzberg SL. The genome polishing tool POLCA makes fast and accurate corrections in genome assemblies. *Plos Comput Biol* 2020;16:e1007981.

16. Seemann T. Snippy: Rapid haploid variant calling and core SNP phylogeny. 2020. https://github.com/tseemann/snippy. Accessed October 11, 2020, 2020.

17. Gouliouris T, Coll F, Ludden C, et al. Quantifying acquisition and transmission of Enterococcus faecium using genomic surveillance. *Nat Microbiol* 2021;6:103-111.

18. Darling AE, Mau B, Perna NT. progressiveMauve: multiple genome alignment with gene gain, loss and rearrangement. *Plos One* 2010;5:e11147.

Supp. Fig. 1 – *Enterococcus faecium* clinical bloodstream isolate collection

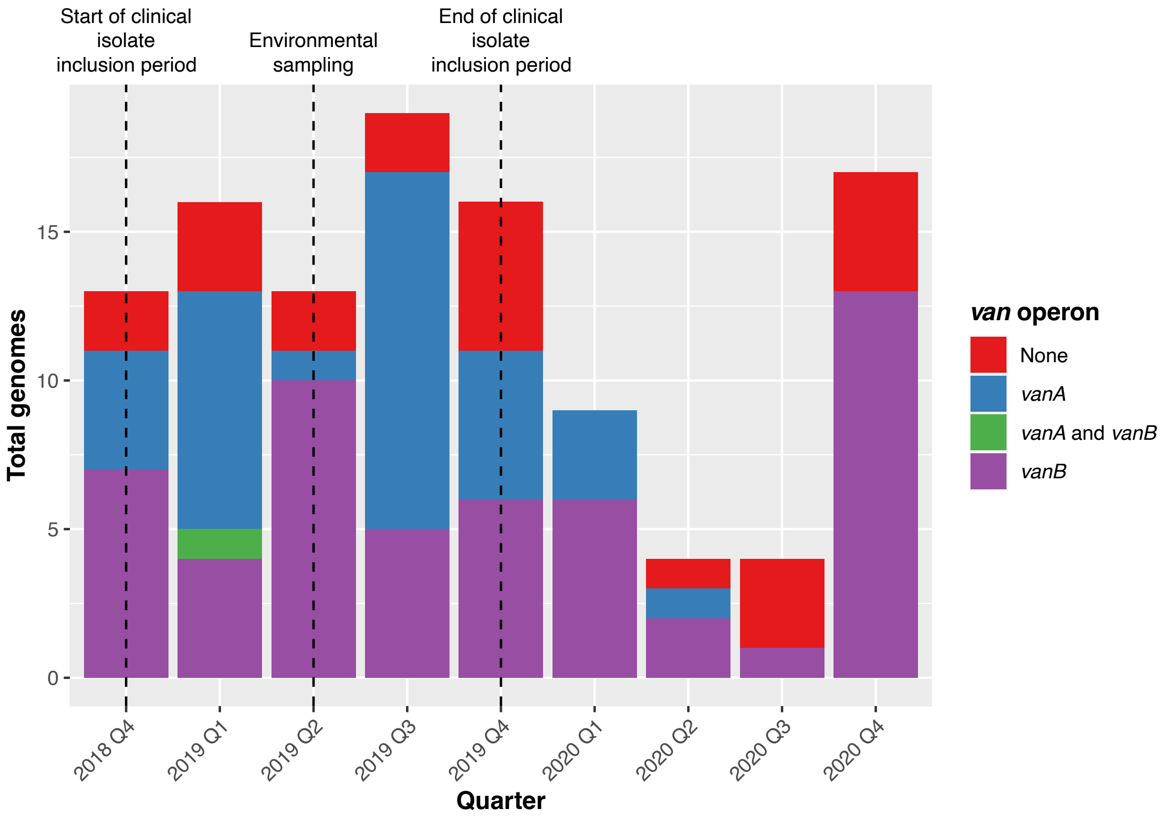

Summary of *E. faecium* bloodstream isolate collection by year and quarter, with presence of *van* operon indicated. Dotted lines show bloodstream isolate inclusion period and time of environmental sampling.

Supp. Fig. 2 – Summary of *Enterococcus faecium* environmental and clinical genomes by ward

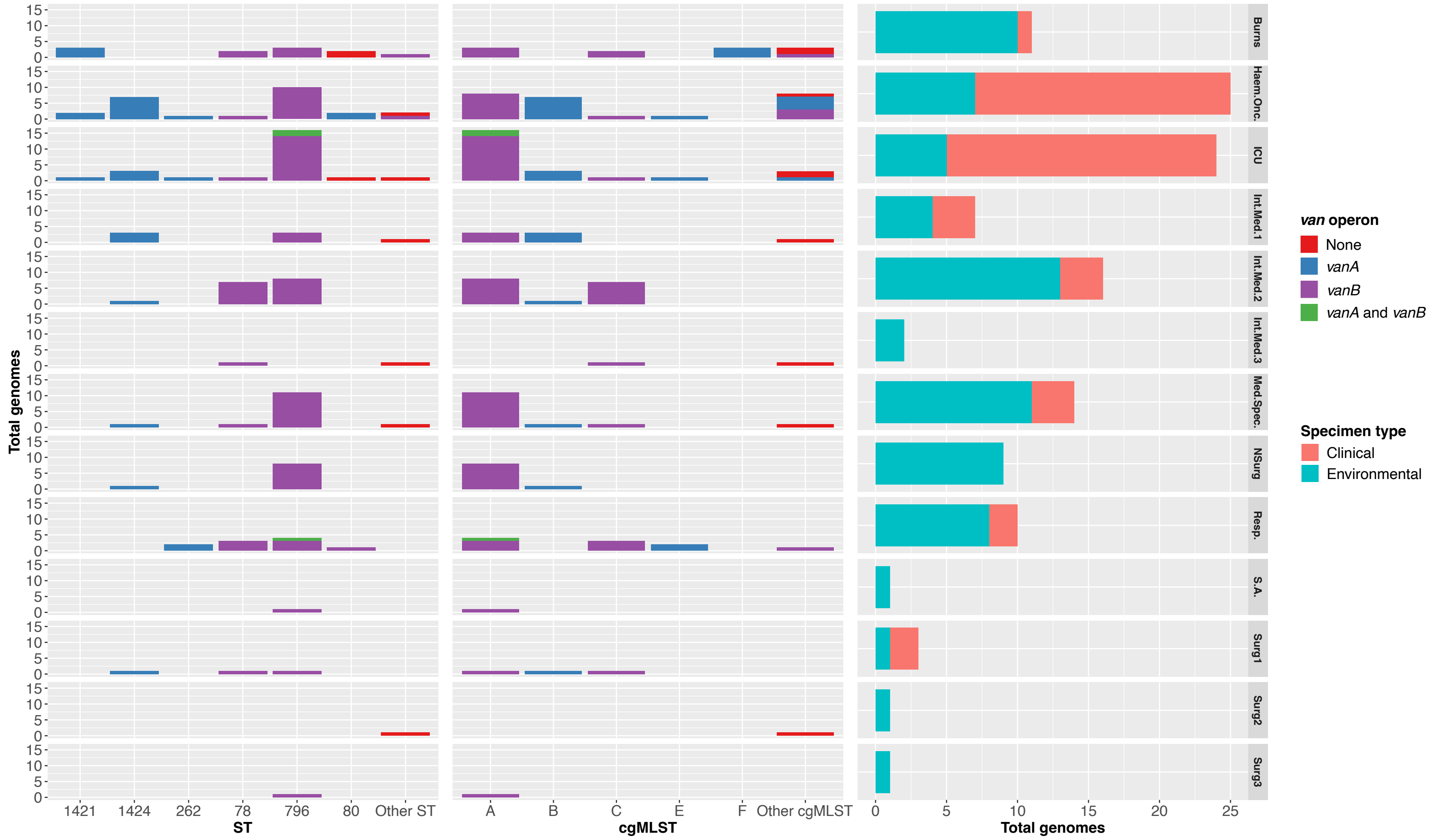

For each ward, environmental and clinical *E. faecium* genomes are shown by multi-locus sequence type (MLST) and core genome MLST (cgMLST). Abbreviations: Haem.Onc. – Haematology/Oncology; ICU – Intensive Care Unit; Int.Med. – Internal Medicine; Med.Spec. – Medical Specialties; NSurg – Neurosurgery; Resp. – Respiratory; S.A. – Subacute; Surg. – Surgery.

Supp. Fig. 3 – Time between collection dates of genetically-related isolates

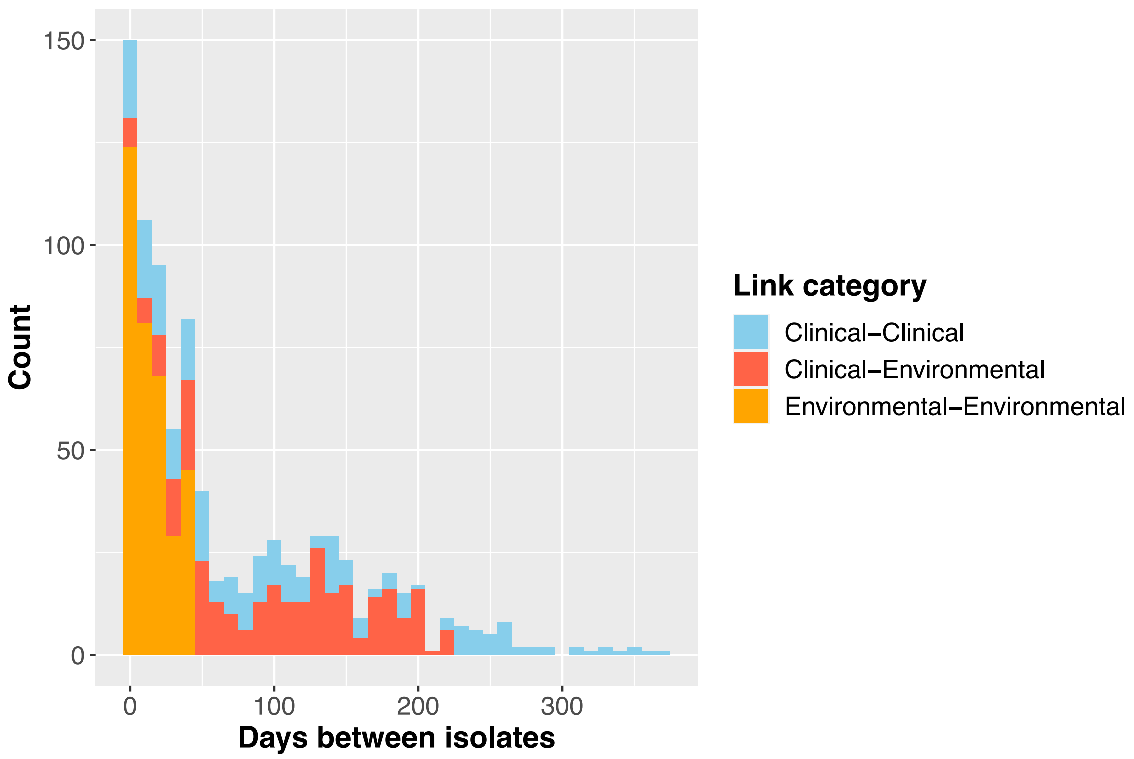

Histogram showing distribution of time difference in collection dates between pairs of genetically related *Enterococcus faecium* isolates (defined as single nucleotide variant distance ≤6).

Supp. Fig. 4 – Plasmids with *van* operons from *Enterococcus faecium* completed assemblies

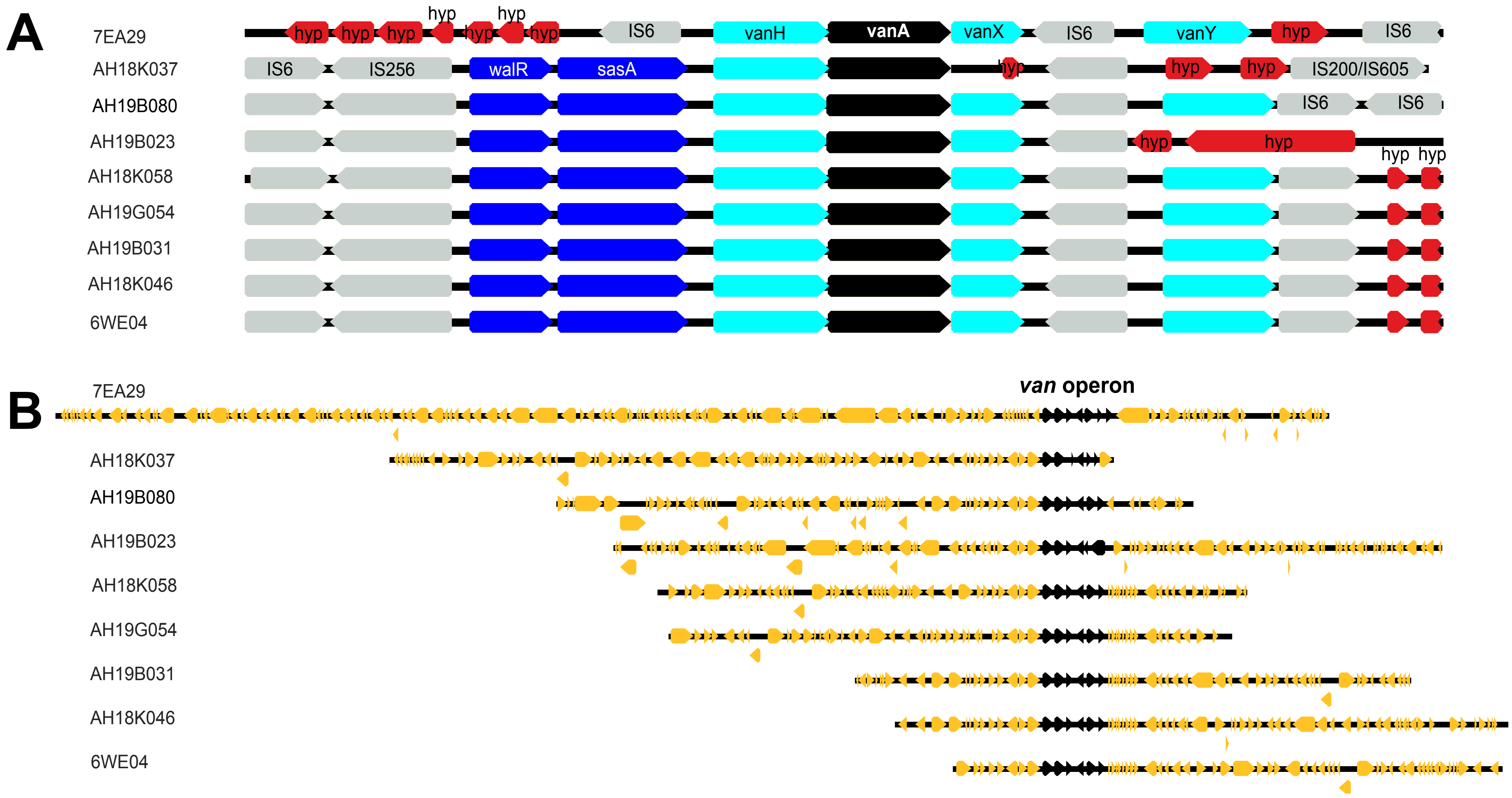

A) Detailed view of *van* operons within Tn1546 transposons from plasmids in completed *E. faecium* assemblies. Isolate numbers are shown in column on left. B) *van* operons (in black) shown in context of entire plasmids (yellow) from completed *E. faecium* assemblies. The plasmids carried additional resistance determinants including *aphA* (aminoglycosides), *erm* (macrolides), *tet(L)* and *tet(M)* (tetracyclines) and *bcrABR* (bacitracin).

Supp. Table 1 - Summary of included clinical and environmental Enterococcus faecium genomes

Isolate	Collection Date	Specimen Type	*van* operon presence	ST	cgMLST cluster	SRA (Illumina) accession no.	SRA (Nanopore) accession no.	GenBank accession no.
2EA01	12/6/2019	Environmental	VanB	796	A	SRR28039592		JBAKTT000000000
2EA04	12/6/2019	Environmental	VanA	1424	B	SRR28039591		JBAKTS000000000
2EA05	12/6/2019	Environmental	VanB	796	A	SRR28039575		JBAKTR000000000
2EA09	12/6/2019	Environmental	VanB	796	A	SRR28039564		JBAKTQ000000000
2EA15	12/6/2019	Environmental	VanB	796	A	SRR28039451		JBAKTP000000000
2EA17	12/6/2019	Environmental	VanB	796	A	SRR28039440		JBAKTO000000000
2EA21	12/6/2019	Environmental	VanB	796	A	SRR28039429		JBAKTN000000000
2EA24	12/6/2019	Environmental	VanB	796	A	SRR28039520		JBAKTM000000000
2EA32	12/6/2019	Environmental	VanB	796	A	SRR28039509		JBAKTL000000000
2WE08	6/5/2019	Environmental	VanB	78	C	SRR28039498		JBAKTK000000000
2WE22B	6/5/2019	Environmental	None	17	D	SRR28039590	SRR28039475	JBAKTJ000000000
2WE35	6/5/2019	Environmental	VanB	796	A	SRR28039546		JBBDHB000000000
4EA04	19/6/2019	Environmental	VanA	1424	B	SRR28039535		JBAKTI000000000
4EA05	19/6/2019	Environmental	VanB	796	A	SRR28039524		JBAKTH000000000
4EA30	19/6/2019	Environmental	VanB	796	A	SRR28039481		JBAKTG000000000
4EA31	19/6/2019	Environmental	VanA	1424	B	SRR28039470		JBAKTF000000000
4WA01	6/5/2019	Environmental	VanB	796	A	SRR28039459		JBAKTE000000000
4WA02	6/5/2019	Environmental	VanB	796	A	SRR28039579		JBAKTD000000000
4WA03	6/5/2019	Environmental	VanB	78	C	SRR28039577		JBAKTC000000000
4WA06	6/5/2019	Environmental	VanB	796	A	SRR28039576		JBAKTB000000000
4WA07	6/5/2019	Environmental	VanB	796	A	SRR28039574		JBAKTA000000000
4WA08	6/5/2019	Environmental	VanB	796	A	SRR28039573		JBAKSZ000000000
4WA09	6/5/2019	Environmental	VanB	796	A	SRR28039572		JBAKSY000000000
4WA10	6/5/2019	Environmental	VanB	78	C	SRR28039571		JBAKSX000000000
4WA14	6/5/2019	Environmental	VanB	78	C	SRR28039570		JBAKSW000000000
4WA16	6/5/2019	Environmental	VanB	796	A	SRR28039569		JBAKSV000000000

4WA17	6/5/2019	Environmental	VanB	78	C	SRR28039568		JBAKSU000000000
4WA28B	6/5/2019	Environmental	VanB	78	C	SRR28039567		JBAKST000000000
4WA31	6/5/2019	Environmental	VanB	78	C	SRR28039566		JBAKSS000000000
5EA26	29/4/2019	Environmental	VanB	78	C	SRR28039565		JBAKSR000000000
5EA27	29/4/2019	Environmental	VanA	262	E	SRR28039563		JBAKSQ000000000
5EA29	29/4/2019	Environmental	VanA	262	E	SRR28039562		JBAKSP000000000
5EA30	29/4/2019	Environmental	VanB	78	C	SRR28039561		JBAKSO000000000
5EA31	29/4/2019	Environmental	VanA and VanB	796	A	SRR28039560		JBAKSN000000000
5EA32	29/4/2019	Environmental	VanB	78	C	SRR28039559		JBAKSM000000000
5EA33	29/4/2019	Environmental	VanB	796	A	SRR28039558		JBAKSL000000000
5EA34	29/4/2019	Environmental	VanB	796	A	SRR28039557		JBAKSK000000000
6WE04	6/5/2019	Environmental	VanA	1421	F	SRR28039556	SRR28039474	JBAKSJ000000000
6WE05	6/5/2019	Environmental	VanB	78	C	SRR28039453		JBAKSI000000000
6WE11	6/5/2019	Environmental	None	80	G	SRR28039452	SRR28039473	JBAKSH000000000
6WE17	6/5/2019	Environmental	VanA	1421	F	SRR28039450		JBAKSG000000000
6WE18A	6/5/2019	Environmental	None	80	G	SRR28039449		JBAKSF000000000
6WE28	6/5/2019	Environmental	VanA	1421	F	SRR28039448		JBAKSE000000000
6WE29	6/5/2019	Environmental	VanB	78	C	SRR28039447		JBAKSD000000000
6WE32	6/5/2019	Environmental	VanB	796	A	SRR28039446		JBAKSC000000000
6WE35	6/5/2019	Environmental	VanB	796	A	SRR28039445		JBAKSB000000000
6WE36A	6/5/2019	Environmental	VanB	555	H	SRR28039444	SRR28039472	JBAKSA000000000
7EA02	6/5/2019	Environmental	VanB	796	A	SRR28039443		JBAKRZ000000000
7EA05	6/5/2019	Environmental	VanB	796	A	SRR28039442		JBAKRY000000000
7EA12	6/5/2019	Environmental	VanB	796	A	SRR28039441		JBAKRX000000000
7EA19	6/5/2019	Environmental	VanA	1424	B	SRR28039439		JBAKRW000000000
7EA29	6/5/2019	Environmental	VanA	80	I	SRR28039438	SRR28039471	JBAKRV000000000
7EA30	6/5/2019	Environmental	VanA	80	I	SRR28039437		JBAKRU000000000
7EA35	6/5/2019	Environmental	VanB	78	C	SRR28039436		JBAKRT000000000
7WE01	29/5/2019	Environmental	VanB	796	A	SRR28039435		JBAKRS000000000
7WE02	29/5/2019	Environmental	VanB	796	A	SRR28039434		JBAKRR000000000

7WE03	29/5/2019	Environmental	VanB	796	A	SRR28039433		JBBDHA000000000
7WE09	29/5/2019	Environmental	VanB	796	A	SRR28039432		JBAKRQ000000000
7WE11	29/5/2019	Environmental	VanB	796	A	SRR28039431		JBAKRP000000000
7WE12	29/5/2019	Environmental	VanB	796	A	SRR28039430		JBAKRO000000000
7WE13	29/5/2019	Environmental	VanB	796	A	SRR28039428		JBAKRN000000000
7WE14	29/5/2019	Environmental	VanB	796	A	SRR28039427		JBAKRM000000000
7WE15	29/5/2019	Environmental	VanB	796	A	SRR28039426		JBAKRL000000000
7WE16	29/5/2019	Environmental	VanB	796	A	SRR28039425		JBAKRK000000000
7WE19	29/5/2019	Environmental	VanB	796	A	SRR28039424		JBAKRJ000000000
AH18K015	7/11/2018	Clinical	VanB	796	A	SRR28039423	SRR28039469	JBAKRI000000000
AH18K037	11/11/2018	Clinical	VanA	1424	B	SRR28039422	SRR28039468	JBAKRH000000000
AH18K046	14/11/2018	Clinical	VanA	1421	Q	SRR28039523	SRR28039467	JBAKRG000000000
AH18K058	19/11/2018	Clinical	VanA	262	E	SRR28039522	SRR28039466	JBAKRF000000000
AH18L008	6/12/2018	Clinical	VanA	1424	B	SRR28039521		JBAKRE000000000
AH18L019	9/12/2018	Clinical	VanB	796	A	SRR28039519		JBAKRD000000000
AH18L053	19/12/2018	Clinical	None	1489	R	SRR28039518	SRR28039465	JBAKRC000000000
AH18L071	26/12/2018	Clinical	VanB	78	C	SRR28039517	SRR28039464	JBAKRB000000000
AH19A002	31/12/2018	Clinical	VanB	796	A	SRR28039516		JBAKRA000000000
AH19A045	12/1/2019	Clinical	None	192	S	SRR28039515	SRR28039463	JBAKQZ000000000
AH19A061	17/1/2019	Clinical	VanB	796	A	SRR28039514		JBAKQY000000000
AH19A067	21/1/2019	Clinical	None	1424	B	SRR28039513		JBAKQX000000000
AH19A086	25/1/2019	Clinical	VanB	796	A	SRR28039512		JBAKQW000000000
AH19B009	6/2/2019	Clinical	VanB	796	A	SRR28039511		JBAKQV000000000
AH19B023	10/2/2019	Clinical	VanA	1421	L	SRR28039510	SRR28039462	JBAKQU000000000
AH19B031	12/2/2019	Clinical	VanA	1421	T	SRR28039508	SRR28039461	JBAKQT000000000
AH19B056	20/2/2019	Clinical	VanA	262	E	SRR28039507		JBAKQS000000000
AH19B080	26/2/2019	Clinical	VanA	1421	J	SRR28039506	SRR28039460	JBAKQR000000000
AH19C003	1/3/2019	Clinical	VanA and VanB	796	A	SRR28039505		JBAKQQ000000000
AH19C007	2/3/2019	Clinical	VanA and VanB	796	A	SRR28039504		JBAKQP000000000
AH19C045	11/3/2019	Clinical	VanA	1424	B	SRR28039503		JBAKQO000000000

AH19C052	14/3/2019	Clinical	VanA	1421	J	SRR28039502		JBAKQN000000000
AH19C054	14/3/2019	Clinical	VanA	1424	B	SRR28039501		JBAKQM000000000
AH19C059	16/3/2019	Clinical	VanA	1424	B	SRR28039500		JBAKQL000000000
AH19C096	29/3/2019	Clinical	None	252	U	SRR28039499	SRR28039458	JBAKQK000000000
AH19C103	31/3/2019	Clinical	VanB	78	C	SRR28039497		JBAKQJ000000000
AH19D045	19/4/2019	Clinical	None	80	V	SRR28039496	SRR28039588	JBAKQI000000000
AH19D049	20/4/2019	Clinical	VanB	252	K	SRR28039495	SRR28039587	JBAKQH000000000
AH19D061	22/4/2019	Clinical	VanB	796	A	SRR28039494		JBAKQG000000000
AH19E019	9/5/2019	Clinical	VanB	796	A	SRR28039493		JBAKQF000000000
AH19E044	21/5/2019	Clinical	VanA	1421	L	SRR28039492		JBAKQE000000000
AH19E068	27/5/2019	Clinical	VanB	78	C	SRR28039457		JBAKQD000000000
AH19F003	1/6/2019	Clinical	None	1424	W	SRR28039456	SRR28039586	JBAKQC000000000
AH19F005	2/6/2019	Clinical	VanB	796	A	SRR28039455		JBAKQB000000000
AH19F029	7/6/2019	Clinical	VanB	80	M	SRR28039454	SRR28039585	JBAKQA000000000
AH19F040	12/6/2019	Clinical	VanB	796	A	SRR28039589		JBAKPZ000000000
AH19F067	25/6/2019	Clinical	VanB	796	N	SRR28039555	SRR28039584	JBAKPY000000000
AH19F070	26/6/2019	Clinical	VanB	796	N	SRR28039554		JBAKPX000000000
AH19F071	26/6/2019	Clinical	VanB	796	A	SRR28039553		JBAKPW000000000
AH19F078	27/6/2019	Clinical	VanB	796	A	SRR28039552		JBAKPV000000000
AH19G039	13/7/2019	Clinical	VanB	796	A	SRR28039551		JBAKPU000000000
AH19G046	16/7/2019	Clinical	VanB	796	A	SRR28039550		JBAKPT000000000
AH19G054	21/7/2019	Clinical	VanA	1425	O	SRR28039549	SRR28039583	JBAKPS000000000
AH19G064	25/7/2019	Clinical	VanA	1424	B	SRR28039548		JBAKPR000000000
AH19G077	29/7/2019	Clinical	VanA	1424	B	SRR28039547		JBAKPQ000000000
AH19G079	30/7/2019	Clinical	VanA	1424	B	SRR28039545		JBAKPP000000000
AH19H004	2/8/2019	Clinical	VanA	1424	B	SRR28039544		JBAKPO000000000
AH19H005	3/8/2019	Clinical	VanA	1424	B	SRR28039543		JBAKPN000000000
AH19H014	6/8/2019	Clinical	VanA	1424	B	SRR28039542		JBAKPM000000000
AH19H029	10/8/2019	Clinical	VanB	796	A	SRR28039541		JBAKPL000000000
AH19H030	11/8/2019	Clinical	VanA	1424	B	SRR28039540		JBAKPK000000000

AH19H058	21/8/2019	Clinical	None	1283	X	SRR28039539	SRR28039582	JBAKPJ000000000
AH19H062	22/8/2019	Clinical	VanA	1424	B	SRR28039538		JBAKPI000000000
AH19H067	24/8/2019	Clinical	None	212	Y	SRR28039537	SRR28039581	JBAKPH000000000
AH19H074	27/8/2019	Clinical	VanA	1424	B	SRR28039536		JBAKPG000000000
AH19H083	29/8/2019	Clinical	VanB	796	A	SRR28039534		JBAKPF000000000
AH19I021	10/9/2019	Clinical	VanA	1424	B	SRR28039533		JBAKPE000000000
AH19I027	11/9/2019	Clinical	VanB	796	A	SRR28039532		JBAKPD000000000
AH19I041	18/9/2019	Clinical	VanA	1424	B	SRR28039531		JBAKPC000000000
AH19I049	20/9/2019	Clinical	VanA	1424	B	SRR28039530		JBAKPB000000000
AH19I064	25/9/2019	Clinical	VanB	796	A	SRR28039529		JBAKPA000000000
AH19J002	2/10/2019	Clinical	VanB	796	A	SRR28039528		JBAKOZ000000000
AH19J027	17/10/2019	Clinical	VanB	78	C	SRR28039527		JBAKOY000000000
AH19J038	20/10/2019	Clinical	VanA	1424	B	SRR28039526		JBAKOX000000000
AH19K007	2/11/2019	Clinical	None	1425	O	SRR28039525		JBAKOW000000000
AH19K008	4/11/2019	Clinical	None	1424	B	SRR28039491		JBAKOV000000000
AH19K011	4/11/2019	Clinical	VanA	1424	B	SRR28039490		JBAKOU000000000
AH19K039	12/11/2019	Clinical	VanB	796	A	SRR28039489		JBAKOT000000000
AH19K042	14/11/2019	Clinical	VanB	78	C	SRR28039488		JBAKOS000000000
AH19K055	18/11/2019	Clinical	VanA	1424	B	SRR28039487		JBAKOR000000000
AH19K073	25/11/2019	Clinical	VanA	1424	B	SRR28039486		JBAKOQ000000000
AH19L034	13/12/2019	Clinical	None	1913	Z	SRR28039485	SRR28039580	JBAKOP000000000
Bed01	6/5/2019	Environmental	None	696	P	SRR28039484	SRR28039578	JBAKOO000000000
Bed08	6/5/2019	Environmental	VanB	78	C	SRR28039483		JBAKON000000000
FFH15	21/5/2019	Environmental	VanB	796	A	SRR28039482		JBBDGZ000000000
ICU20	13/5/2019	Environmental	VanB	796	A	SRR28039480		JBAKOM000000000
ICU55	13/5/2019	Environmental	VanB	796	A	SRR28039479		JBAKOL000000000
ICU56	13/5/2019	Environmental	VanB	796	A	SRR28039478		JBAKOK000000000
ICU61A	13/5/2019	Environmental	VanB	796	A	SRR28039477		JBAKOJ000000000
ICU64	13/5/2019	Environmental	VanB	796	A	SRR28039476		JBAKOI000000000

Supp. Table 2 – Environmental surface types sampled during study

Surface type
Computer station
Blood pressure machine
Glucometer
Thermometer
Commode
Mattress
Chair
Trolley
Bed side monitors
Other

Supp. Table 3 – Summary of environmental swabs positive for vancomycin-resistant *Enterococcus faecium* by ward

Ward	Proportion of swabs positive	Standard error
Neurosurgery	0.3	0.08
Surgical 1	0.12	0.06
Surgical 2	0	0
Internal Medicine 1	0.13	0.06
Internal Medicine 2	0.57	0.10
Respiratory	0.23	0.07
Burns	1.0	0
Haematology/Oncology	0.20	0.07
Medical Specialties	0.32	0.08
Internal Medicine 3	0.20	0.13
Subacute	0.05	0.05
ICU	0.10	0.04

Supp. Table 4 – Summary of surface types positive for vancomycin-resistant *Enterococcus faecium*

Surface type	Proportion of swabs positive	Standard error
Blood pressure machine	0.15	0.05
Patient chair	0.52	0.10
Commode	0.38	0.11
Glucometer	0.15	0.07
Mattress	0.24	0.07
Other	0.21	0.08
Computer station	0.19	0.03
Thermometer	0.04	0.03

Supp. Table 5 – Summary of putative *Enterococcus faecium* genomic transmission clusters

Putative genomic transmission cluster	Total genomes	Clinical	Environmental	*vanA*	*vanB*	*vanA* and *vanB*	No *van* operon
1	32	9	23	0	31	1	0
2	26	22	4	25	0	0	1
3	14	2	12	0	14	0	0
4	10	3	7	0	10	0	0
5	6	2	4	0	6	0	0
6	4	0	4	0	4	0	0
7	3	0	3	3	0	0	0
8	2	1	1	0	2	0	0
9	2	1	1	0	2	0	0
10	2	2	0	0	0	2	0
11	2	2	0	2	0	0	0
12	2	2	0	0	2	0	0
13	2	2	0	1	0	0	1
14	2	2	0	2	0	0	0
